# Pelvic trauma: WSES classification and guidelines

**DOI:** 10.1186/s13017-017-0117-6

**Published:** 2017-01-18

**Authors:** Federico Coccolini, Philip F. Stahel, Giulia Montori, Walter Biffl, Tal M Horer, Fausto Catena, Yoram Kluger, Ernest E. Moore, Andrew B. Peitzman, Rao Ivatury, Raul Coimbra, Gustavo Pereira Fraga, Bruno Pereira, Sandro Rizoli, Andrew Kirkpatrick, Ari Leppaniemi, Roberto Manfredi, Stefano Magnone, Osvaldo Chiara, Leonardo Solaini, Marco Ceresoli, Niccolò Allievi, Catherine Arvieux, George Velmahos, Zsolt Balogh, Noel Naidoo, Dieter Weber, Fikri Abu-Zidan, Massimo Sartelli, Luca Ansaloni

**Affiliations:** 1 General, Emergency and Trauma Surgery, Papa Giovanni XXIII Hospital, P.zza OMS 1, 24128 Bergamo, Italy; 2 Department of Orthopedic Surgery and Department of Neurosurgery, Denver Health Medical Center and University of Colorado School of Medicine, Denver, CO USA; 3 Acute Care Surgery, The Queen’s Medical Center, Honolulu, HI USA; 4 Dept. of Cardiothoracic and Vascular Surgery & Dept. Of Surgery Örebro University Hospital and Örebro University, Örebro, Sweden; 5 Emergency and Trauma Surgery, Maggiore Hospital, Parma, Italy; 6 Division of General Surgery Rambam Health Care Campus Haifa, Haifa, Israel; 7 Trauma Surgery, Denver Health, Denver, CO USA; 8 Surgery Department, University of Pittsburgh, Pittsburgh, Pensylvania USA; 9 Virginia Commonwealth University, Richmond, VA USA; 10 Department of Surgery, UC San Diego Health System, San Diego, USA; 11 Faculdade de Ciências Médicas (FCM) – Unicamp, Campinas, SP Brazil; 12 Trauma & Acute Care Service, St Michael’s Hospital, Toronto, ON Canada; 13 General, Acute Care, Abdominal Wall Reconstruction, and Trauma Surgery Foothills Medical Centre, Calgary, AB Canada; 14 Abdominal Center, University Hospital Meilahti, Helsinki, Finland; 15 Emergency and Trauma Surgery, Niguarda Hospital, Milan, Italy; 16 Digestive and Emergency Surgery, UGA-Université Grenoble Alpes, Grenoble, France; 17 Harvard Medical School, Division of Trauma, Emergency Surgery and Surgical Critical Care Massachusetts General Hospital, Boston, MA USA; 18 Department of Traumatology, John Hunter Hospital and University of Newcastle, Newcastle, NSW Australia; 19 Department of Surgery, University of KwaZulu-Natal, Durban, South Africa; 20 Department of General Surgery, Royal Perth Hospital, Perth, Australia; 21 Department of Surgery, College of Medicine and Health Sciences, UAE University, Al-Ain, United Arab Emirates; 22 General and Emergency Surgery, Macerata Hospital, Macerata, Italy

**Keywords:** Pelvic, Trauma, Management, Guidelines, Mechanic, Injury, Angiography, REBOA, ABO, Preperitoneal pelvic packing, External fixation, Internal fixation, X-ray, Pelvic ring fractures

## Abstract

Complex pelvic injuries are among the most dangerous and deadly trauma related lesions. Different classification systems exist, some are based on the mechanism of injury, some on anatomic patterns and some are focusing on the resulting instability requiring operative fixation. The optimal treatment strategy, however, should keep into consideration the hemodynamic status, the anatomic impairment of pelvic ring function and the associated injuries. The management of pelvic trauma patients aims definitively to restore the homeostasis and the normal physiopathology associated to the mechanical stability of the pelvic ring. Thus the management of pelvic trauma must be multidisciplinary and should be ultimately based on the physiology of the patient and the anatomy of the injury. This paper presents the World Society of Emergency Surgery (WSES) classification of pelvic trauma and the management Guidelines.

## Background

Pelvic trauma (PT) is one of the most complex management in trauma care and occurs in 3% of skeletal injuries [1–4]. Patients with pelvic fractures are usually young and they have a high overall injury severity score (ISS) (25 to 48 ISS) [3]. Mortality rates remain high, particularly in patients with hemodynamic instability, due to the rapid exsanguination, the difficulty to achieve hemostasis and the associated injuries [1, 2, 4, 5]. For these reasons, a multidisciplinary approach is crucial to manage the resuscitation, to control the bleeding and to manage bones injuries particularly in the first hours from trauma. PT patients should have an integrated management between trauma surgeons, orthopedic surgeons, interventional radiologists, anesthesiologists, ICU doctors and urologists 24/7 [6, 7].

At present no comprehensive guidelines have been published about these issues. No correlation has been demonstrated to exist between type of pelvic ring anatomical lesions and patient physiologic status. Moreover the management of pelvic trauma has markedly changed throughout the last decades with a significant improvement in outcomes, due to improvements in diagnostic and therapeutic tools. In determining the optimal treatment strategy, the anatomical lesions classification should be supplemented by hemodynamic status and associated injuries. The anatomical description of pelvic ring lesions is fundamental in the management algorithm but not definitive. In fact, in clinical practice the first decisions are based mainly on the clinical conditions and the associated injuries, and less on the pelvic ring lesions. Ultimately, the management of trauma requires an assessment of the anatomical injury and its physiologic effects.

This paper aims to present the World Society of Emergency Surgery (WSES) classification of pelvic trauma and the treatment Guidelines.

WSES includes surgeons from whole world. This Classification and Guidelines statements aim to direct the management of pelvic trauma, acknowledging that there are acceptable alternative management options. In reality, as already considered for other position papers and guidelines, not all trauma surgeons work in the same conditions and have the same facilities and technologies available [8].

### Notes on the use of the guidelines

The Guidelines are evidence-based, with the grade of recommendation also based on the evidence. The Guidelines present the diagnostic and therapeutic methods for optimal management of pelvic trauma. The practice Guidelines promulgated in this work do not represent a standard of practice. They are suggested plans of care, based on best available evidence and the consensus of experts, but they do not exclude other approaches as being within the standard of practice. For example, they should not be used to compel adherence to a given method of medical management, which method should be finally determined after taking account of the conditions at the relevant medical institution (staff levels, experience, equipment, etc.) and the characteristics of the individual patient. However, responsibility for the results of treatment rests with those who are directly engaged therein, and not with the consensus group.

## Methods

Eight specific questions were addressed regarding the management of PT assessing the main problems related to the hemodynamic and the mechanical status:

1. Which are the main diagnostic tools necessary prior to proceed in hemodynamically unstable PT?
2. Which is the role of pelvic binder in hemodynamically unstable pelvic fracture?
3. Which is the role of Resuscitative Endovascular Balloon Occlusion of the Aorta (REBOA) in hemodynamically unstable pelvic trauma?
4. Which patients with hemodynamically unstable PT warrant preperitoneal pelvic packing?
5. Which patients with hemodynamically unstable pelvic ring injuries require external pelvic fixation?
6. Which patients with hemodynamically unstable PT warrant angioembolization?
7. What are the indications for definitive surgical fixation of pelvic ring injuries?
8. What is the ideal time-window to proceed with definitive internal pelvic fixation?

**Fig. 1:**
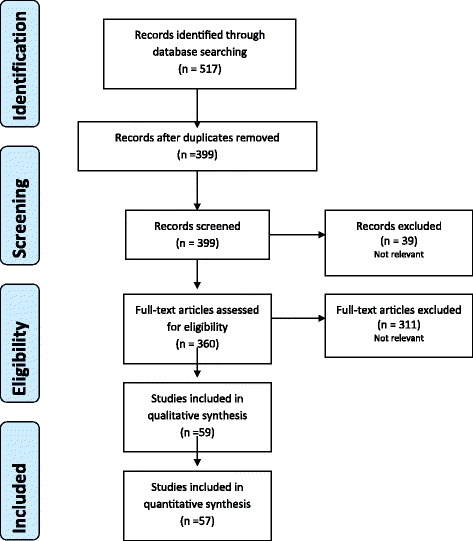
PRISMA flow diagram

A computerized search was done by the bibliographer in different databanks (MEDLINE, SCOPUS, EMBASE) citations were included for the period between January 1980 to December 2015 using the primary search strategy: pelvis, pelvic, injuries, trauma, resuscitation, sacral, bone screws, fractures, external fixation, internal fixation, anterior e posterior fixation, hemodynamic instability/stability, packing, pubic symphisis, angioembolization, pelvic binder/binding, aortic, balloon, occlusion, resuscitative, definitive, stabilization combined with AND/OR. No search restrictions were imposed. The dates were selected to allow comprehensive published abstracts of clinical trials, consensus conference, comparative studies, congresses, guidelines, government publication, multicenter studies, systematic reviews, meta-analysis, large case series, original articles, randomized controlled trials. Case reports and small cases series were excluded. No randomized controlled trials were found. Narrative review articles were also analyzed to determine other possible studies. Literature selection is reported in the flow chart (Fig. 1). The Level of Evidence (LE) was evaluated using the GRADE system [9] (Table 1).

**Table 1:** GRADE system to evaluate the level of evidence and recommendation

Grade of recommendation	Clarity of risk/benefit	Quality of supporting evidence	Implications
1A			
Strong recommendation, high-quality evidence	Benefits clearly outweigh risk and burdens, or vice versa	RCTs without important limitations or overwhelming evidence from observational studies	Strong recommendation, applies to most patients in most circumstances without reservation
1B			
Strong recommendation, moderate-quality evidence	Benefits clearly outweigh risk and burdens, or vice versa	RCTs with important limitations (inconsistent results, methodological flaws, indirect analyses or imprecise conclusions) or exceptionally strong evidence from observational studies	Strong recommendation, applies to most patients in most circumstances without reservation
1C			
Strong recommendation, low-quality or very low-quality evidence	Benefits clearly outweigh risk and burdens, or vice versa	Observational studies or case series	Strong recommendation but subject to change when higher quality evidence becomes available
2A			
Weak recommendation, high-quality evidence	Benefits closely balanced with risks and burden	RCTs without important limitations or overwhelming evidence from observational studies	Weak recommendation, best action may differ depending on the patient, treatment circumstances, or social values
2B			
Weak recommendation, moderate-quality evidence	Benefits closely balanced with risks and burden	RCTs with important limitations (inconsistent results, methodological flaws, indirect or imprecise) or exceptionally strong evidence from observational studies	Weak recommendation, best action may differ depending on the patient, treatment circumstances, or social values
2C			
Weak recommendation, Low-quality or very low-quality evidence	Uncertainty in the estimates of benefits, risks, and burden; benefits, risk, and burden may be closely balanced	Observational studies or case series	Very weak recommendation; alternative treatments may be equally reasonable and merit consideration

The discussion of the present guidelines has been realized through the Delphi process. A group of experts in the field coordinated by a central coordinator was contacted separately to express their evidence-based opinion on the different questions about the hemodynamically and mechanically unstable pelvic trauma management. Pelvic trauma patterns were differentiated into hemodynamically and mechanically stable and unstable ones. Conservative and operative management for all combinations of these conditions were evaluated. The central coordinator assembled the different answers derived from the first round and drafted the first version that was subsequently revised by each member of an enlarged expert group separately. The central coordinator addressed the definitive amendments, corrections and concerns. The definitive version about which the agreement was reached consisted in the published guidelines.

### Mechanisms of injuries

Principal mechanisms of injuries that cause a pelvic ring fracture are due to a high energy impact as fall from height, sports, road traffic collision (pedestrian, motorcyclist, motor vehicle, cyclist), person stuck by vehicles [1, 5]. Ten to fifteen percent of patients with pelvic fractures arrive to the ED in shock and one third of them will die reaching a mortality rate in the more recent reports of 32% [10]. The causes of dying are represented in the major part by uncontrolled bleeding and by patient’s physiologic exhaustion.

### Anatomy of pelvis and pelvic injuries

Pelvic ring is a close compartment of bones containing urogenital organs, rectum, vessels and nerves. Bleeding from pelvic fractures can occur from veins (80%) and from arteries (20%) [7, 11]. Principal veins injured are presacral plexus and prevescical veins, and the principals arteries are anterior branches of the internal iliac artery, the pudendal and the obturator artery anteriorly, and superior gluteal artery and lateral sacral artery posteriorly [7, 11]. Others sources of bleeding include bones fractures [1]. Among the different fracture patterns affecting the pelvic ring each has a different bleeding probability. No definitive association between fracture pattern and bleeding exist but some pattern as APC III are associated to a greater transfusion rate according to some studies [12]. Part of the bleeding is from the bones as clearly showed since 1973. The necessity to fix the bones fractures by repositioning them has been explained by Huittimen et al. [13]. In cases of high-grade injuries, thoraco-abdominal associated injuries can occur in 80%, and others local lesions such as bladder, urethra (1.6-25% of cases), vagina, nerves, sphincters and rectum (18–64%), soft tissues injuries (up to 72%). These injuries should be strongly suspected particularly in patients with perineal hematoma or large soft tissue disruption [1, 3, 14]. These patients need an integrate management with other specialists. Some procedures like supra-pubic catheterization of bladder, colostomy with local debridement and drainage, and antibiotic prevention are important to avoid aggravating urethral injuries or to avoid fecal contamination in case of a digestive tract involvement [1]. Although these conditions must be respected and kept in mind the first aim remains the hemodynamic and pelvic ring stabilization.

### Physiopathology of the injuries

The lesions at the level of the pelvic ring can create instability of the ring itself and a consequent increase in the internal volume. This increase in volume, particular in open book lesions, associated to the soft tissue and vascular disruption, facilitate the increasing hemorrhage in the retroperitoneal space by reducing the tamponing effect (pelvic ring can contain up to a few liters of blood) and can cause an alteration in hemodynamic status [7, 15]. In the management of severely injured and bleeding patients a cornerstone is represented by the early evaluation and correction of the trauma induced coagulopathy. Resuscitation associated to physiologic impairment and to suddenly activation and deactivation of several procoagulant and anticoagulant factors contributes to the insurgence of this frequently deadly condition. The massive transfusion protocol application is fundamental in managing bleeding patients. As clearly demonstrated by the literature blood products, coagulation factors and drugs administration has to be guided by a tailored approach through advanced evaluation of the patient’s coaugulative asset [16–22]. Some authors consider a normal hemodynamic status when the patient does not require fluids or blood to maintain blood pressure, without signs of hypoperfusion; hemodynamic stability as a counterpart is the condition in which the patient achieve a constant or an amelioration of blood pressure after fluids with a blood pressure >90 mmHg and heart rate <100 bpm [23]; hemodynamic instability is the condition in which the patient has an admission systolic blood pressure <90 mmHg, or > 90 mmHg but requiring bolus infusions/transfusions and/or vasopressor drugs and/or admission base deficit (BD) >6 mmol/l and/or shock index > 1 [24, 25] and/or transfusion requirement of at least 4–6 Units of packed red blood cells within the first 24 hours [5, 16, 26]. The Advanced Trauma Life Support (ATLS) definition considers as “unstable” the patient with: blood pressure < 90 mmHg and heart rate > 120 bpm, with evidence of skin vasoconstriction (cool, clammy, decreased capillary refill), altered level of consciousness and/or shortness of breath [26]. The present classification and guideline utilize the ATLS definition. Some authors suggested that the sacroiliac joint disruption, female gender, duration of hypotension, an hematocrit of 30% or less, pulse rate of 130 or greater, displaced obturator ring fracture, a pubic symphysis diastasis can be considered good predictors of major pelvic bleeding [2, 15, 27]. However unfortunately the extent of bleeding is not always related with the type of lesions and there is a poor correlation between the grade of the radiological lesions and the need for emergent hemostasis [7, 15, 28].

### WSES Classification

The anatomical description of pelvic ring lesions is not definitive in the management of pelvic injuries. The classification of pelvic trauma into minor, moderate and severe considers the pelvic ring injuries anatomic classification (Antero-Posterior Compression APC; Lateral Compression LC; Vertical Shear VS; CM: Combined Mechanisms) and more importantly, the hemodynamic status. As already stated the ATLS definition considers as “unstable” the patient with: blood pressure < 90 mmHg and heart rate > 120 bpm, with evidence of skin vasoconstriction (cool, clammy, decreased capillary refill), altered level of consciousness and/or shortness of breath [26].

The WSES Classification divides Pelvic ring Injuries into three classes:

- ***Minor*** (WSES grade I) comprising hemodynamically and mechanically stable lesions
- ***Moderate*** (WSES grade II, III) comprising hemodynamically stable and mechanically unstable lesions
- ***Severe*** (WSES grade IV) comprising hemodynamically unstable lesions independently from mechanical status.

**Fig. 2:**
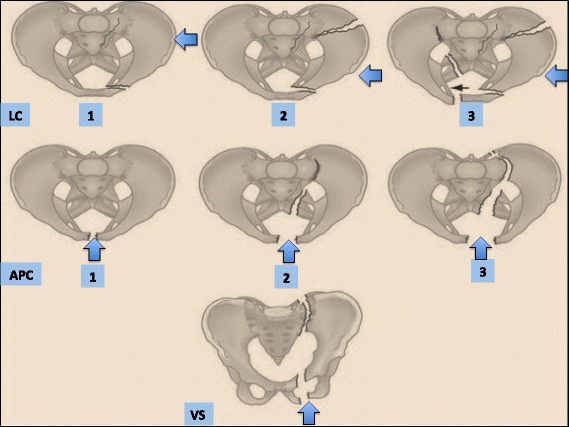
Young and Burgees classification for skeletal pelvic lesions

The classification (Table 2) considers the Young-Burgees classification (Fig. 2), the hemodynamic status and the associated lesions.

**Table 2:** WSES pelvic injuries classification (*: patients hemodynamically stable and mechanically unstable with no other lesions requiring treatment and with a negative CT-scan, can proceed directly to definitive mechanical stabilization. LC: Lateral Compression, APC: Antero-posterior Compression, VS: Vertical Shear, CM: Combined Mechanism, NOM: Non-Operative Management, OM: Operative Management, REBOA: Resuscitative Endo-Aortic Balloon)

	WSES grade	Young-Burgees classification	Haemodynamic	Mechanic	CT-scan	First-line Treatment
MINOR	WSES grade I	APC I – LC I	Stable	Stable	Yes	NOM
MODERATE	WSES grade II	LC II/III - APC II/III	Stable	Unstable	Yes	Pelvic Binder in the field ± Angioembolization (if blush at CT-scan) OM – Anterior External Fixation *
	WSES grade III	VS - CM	Stable	Unstable	Yes	Pelvic Binder in the field ± Angioembolization (if blush at CT-scan) OM - C-Clamp *
SEVERE	WSES grade IV	Any	Unstable	Any	No	Pelvic Binder in the field Preperitoneal Pelvic Packing ± Mechanical fixation (see over) ± REBOA ± Angioembolization

*Minor pelvic injuries:*

- *WSES grade I (should be formatted in bold and cursive as the other grade of classification)* includes APC I, LC I hemodynamically stable pelvic ring injuries.

*Moderate pelvic injuries:*

- ***WSES grade II*** includes APC II – III and LC II - III hemodynamically stable pelvic ring injuries.
- ***WSES grade III*** includes VS and CM hemodynamically stable pelvic ring injuries.

*Severe pelvic injuries:*

- ***WSES grade IV*** includes any hemodynamically unstable pelvic ring injuries.

**Fig. 3:**
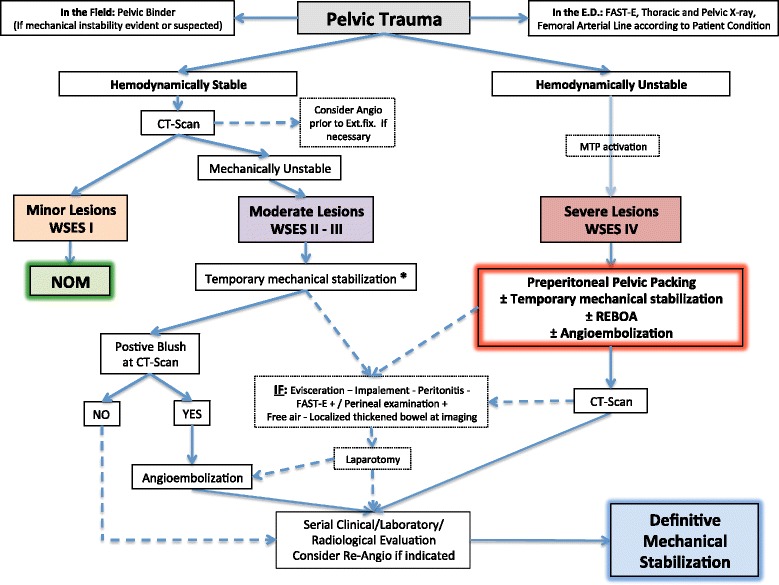
Pelvic Trauma management algorithm (*: patients hemodynamically stable and mechanically unstable with no other lesions requiring treatment and with a negative CT-scan, can proceed directly to definitive mechanical stabilization. MTP: Massive Transfusion Protocol, FAST-E: Eco-FAST Extended, ED: Emergency Department, CT: Computed Tomography, NOM: Non Operative Management, HEMODYNAMIC STABILITY is the condition in which the patient achieve a constant or an amelioration of blood pressure after fluids with a blood pressure >90 mmHg and heart rate <100 bpm; HEMODYNAMIC INSTABILITY is the condition in which the patient has an admission systolic blood pressure <90 mmHg, or > 90 mmHg but requiring bolus infusions/transfusions and/or vasopressor drugs, or admission base deficit (BD) >6 mmol/l, or shock index > 1, or transfusion requirement of at least 4–6 Units of packed red blood cells within the first 24 h)

Basing on the present classification WSES indicates a management algorithm explained in Fig. 3.

### Principles and cornerstones of the management

The management of pelvic trauma as for all the other politraumatized patients needs to pose in definitive the attention in treating also the physiology; decisions can be more effective when combining evaluation of anatomy, mechanical consequences of injury and their physiological effects. During daily clinical practice the first decisions are based mainly on the clinical conditions and the associated injuries, and less on the pelvic ring lesions. The management of trauma in fact aims firstly to restore the altered physiology. The main aims of proper PT management are bleeding control and stabilization of the hemodynamic status, restoring of the eventual coagulation disorders and the mechanical integrity and stability of the pelvic ring, and preventing complications (septic, urogenital, intestinal, vascular, sexual functions, walking) (×9); then to definitively stabilize the pelvis.

### Recommendations for diagnostic tools use in Pelvic Trauma

- *The time between arrival in the Emergency Department and definitive bleeding control should be minimized to improve outcomes of patients with hemodynamically unstable pelvic fractures [Grade 2A].*
*- Serum lactate and base deficit represent sensitive diagnostic markers to estimate the extent of traumatic-haemorrhagic shock, and to monitor response to resuscitation [Grade 1B].*
*- The use of Pelvic X-ray and E-FAST in the Emergency Department is recommended in hemodynamic and mechanic unstable patients with pelvic trauma and allows to identify the injuries that require an early pelvic stabilization, an early angiography, and a rapid reductive maneuver, as well as laparotomy [Grade 1B].*
*- Patients with pelvic trauma associated to hemodynamic normality or stability should undergo further diagnostic workup with multi phasic CT-scan with intravenous contrast to exclude pelvic hemorrhage [Grade 1B].*
*- CT-scan with 3-Dimensional bones reconstructions reduces the tissue damage during invasive procedures, the risk of neurological disorders after surgical fixation, operative time, and irradiation and the required expertise [Grade 1B].*
*- Retrograde urethrogram or/and urethrocystogram with contrast CT-scan is recommended in presence of local perineal clinical hematoma and pelvic disruption at Pelvic X-ray [Grade 1B].*
*- Perineal and a rectal digital examination are mandatory in case of high suspicious of rectal injuries [Grade 1B].*
*- In case of a positive rectal examination, proctoscopy is recommended [Grade 1C].*

Diagnostic workup strategies in the emergency room must be standardized and streamlined in order to avoid an unnecessary delay to definitive bleeding control, the time between trauma and operating room has been shown to inversely correlate with survival in patients with traumatic pelvic hemorrhage [29].

Sensitive ***laboratory markers*** of acute traumatic hemorrhage include serum lactate and base deficit by arterial blood gas analysis [29]. In contrast, hemoglobin level and hematocrit do not represent sensitive early markers of the extent of traumatic hemorrhagic shock [29]. As coagulopathic patients with traumatic hemorrhagic shock form unstable pelvic ring injuries have a significantly increased post-injury mortality [16], the presence of coagulopathy should be determined early by “point-of-care“ bedside testing using Thromboelastography (TEG) or Rotational Thromboelastometry (ROTEM), which allow targeted resuscitation with blood products and improved post-injury survival rates [17, 19–22]. At first, the evaluation of a PT should be based on the mechanism of injury (particularly in case of high-energy impact, more frequent in blunt trauma) and physical examination to search a pelvic ring deformity or instability, a pelvic or perineal hematoma, or a rectal/urethral bleeding [1]. Lelly maneuver can be useful in evaluating the pelvic ring stability but it should be done cautiously because it can sometime increase the bleeding by dislocating bones margin. In case of hemodynamic instability, particularly in blunt trauma, chest and pelvic x-rays and extended focused assessment for sonographic evaluation of trauma patients (E-FAST) are performed according to ATLS protocols. Chest X-rays and E-FAST are performed to exclude others sours of hemorrhage in the thorax and in the abdomen [1, 7, 30, 31]. The Eastern Association for the Surgery of Trauma guidelines [2] reported that E-FAST is not enough sensitive to exclude a pelvic bleeding, however it could be considered adequate to exclude the need for a laparotomy in unstable patients.

***Pelvic X-ray (PXR)*** in hemodynamically unstable patients helps in identifying life-threatening pelvic ring injuries [18, 32, 33]. It is important but its execution must not delay in proceeding with life-saving maneuvers. Sensitivity and sensibility rates are low (50–68% and 98% respectively) and the false negative rates are high (32%) [23, 34]. For these reason some authors suggested to abandon PXR in case of stable patients [11, 23, 34]. The principal injuries related with hemodynamic instability are sacral fractures, open-book injuries and vertical-shear injuries (APC II-III, LC II-III and VS) [34]. To clearly define injury pattern, it is fundamental to achieve early pelvic stabilization and to early plan for the subsequent diagnostic-therapeutic approach. Moreover PXR is important to evaluate the hip dislocation in order to provide a prompt reductive maneuver [34]. However PXR alone does not predict mortality, hemorrhage or need for angiography [2]. In hemodynamically normal patients with nor pelvic instability nor hip dislocation nor positive physical examination scheduled for CT-scan PXR could be omitted [11].

At the end of primary evaluation a radiological workup is performed. In case of hemodynamic normality or stability ***Computed Tomography (CT)*** is the gold standard with a sensitivity and specificity for bones fractures of 100% [1, 23, 34]. The main two factors that are important to plan a correct decision-making process and to steer the angiography are the presence at CT of intra-venous contrast extravasation and the pelvic hematoma size [2, 35]. CT has an accuracy of 98% for identifying patients with blush, however an absence of blush in contrast CT does not always exclude an active pelvic bleeding [2, 28]. In presence of a pelvic hematoma ≥500 cm3 an arterial injury should be strongly suspected even in absence of a visible contrast blush [2]. CT is useful also to evaluate any injuries of other organs, retroperitoneum, and bones but also to better decide the subsequent surgical management [34]. A recent study supports the use of a multidetector CT with a three phases protocol (arterial, portal and delayed phase) with a subsequent digital subtraction angiography (DSA) in case of suspect of arterial hemorrhage so as to better evaluate bleeding or hematoma [35]. This protocol could significantly reduce the rate of subsequent interventions due to others hemorrhagic foci [35].

***CT with 3-Dimensional bone reconstruction*** is helpful reducing tissue damage during invasive procedure, reducing the subjective expertise required from clinical staff and improving patient recovery times [36]. Chen and coll. reported successful rates of screw positioning in 93.8% of cases after 3D CT reconstruction, particularly in patients with sacral fractures and ilio-sacral joint dislocations [36]. This approach permits to also reduce the neurological disorders after surgical fixation, operative times, and irradiation.

In 7-25% of pelvic ring fractures lower urinary tract and urethra are damaged. However the diagnosis of urethral injuries remains difficult at the initial evaluation and about 23% of them are missed [14]. Clinical signs suggesting a urethral injury are perineal/scrotal hematoma, blood from the urethral meatus, the presence of a high-riding or non-palpable prostate at rectal exploration, the presence of an unstable pelvic fracture. The insertion of a transurethral catheter without other previous investigations in patients with a pelvic injury could be associated with severe complications: either acute like complete transection of the urethra, or chronic like stricture formation, impotence and urinary incontinence [14]. For this reason ATLS guidelines, the World Health Organization and some authors [14] suggested a ***retrograde urethrogram (RUG)*** prior the urethral catheterization. RUGs is recommended when local clinical signs or a disruption in the PXR are found, particularly in the presence of higher degree of soft tissue disruption, bone displacement, or multiple fractures [14]. In case a positive of RUG or when high suspicion of urethral injury are present, a suprapubic catheter with delayed cystogram is recommended [14]. Magnetic resonance images seem promising to detect type of injuries and could be a useful tool in combination with RUGs or in alternative but only in stable patients [14]. However the sequence between RUG and ***urethrocystogram with contrast CT*** is controversial [2]. Performing a RUG before CT could increase the rate of indeterminate and false-negative CT-scans [2]. For this reason when hemodynamic status permits in case of suspected urethral injuries the late contrast CT-scan with a urologic study is recommended [2].

The high incidence of ano-rectal lesions (18–64%) requires careful study of the ano-rectal region. At first a ***perineal and a rectal digital examination*** to detect blood, rectal wall weakness and non-palpable prostate should be done. In case of positive rectal examination a ***rigid proctoscopy*** should be strongly considered [3].

Tile Classification and Young and Burgess Classification (Fig. 2) are the most commonly used classifications for pelvic ring injuries. These classifications are based on the direction of forces causing fracture and the associated instability of pelvis with four injury patterns: lateral compression, antero-posterior compression (external rotation), vertical shear, combined mechanism [12]. The Young and Burgess classification is more beneficial for specialists, as a counterpart the second seems to be more easily remembered and applied.

### Role of pelvic binder in hemodynamically unstable pelvic fractures

- *The application of non-invasive external pelvic compression is recommended as an early strategy to stabilize the pelvic ring and decrease the amount of pelvic haemorrhage in the early resuscitation phase. [Grade 1A]*
*- Pelvic binders are superior to sheet wrapping in the effectiveness of pelvic haemorrhage control [Grade 1C].*
*- Non-invasive external pelvic compression devices should be removed as soon as physiologically justifiable, and replaced by external pelvic fixation, or definitive pelvic stabilization, if indicated [Grade 1B].*
*- Pelvic binders should be positioned cautiously in pregnant women and elderly patients [Grade 2A].*
*- In a patient with pelvic binder whenever it’s possible, an early transfer from the spine board reduces significantly the skin pressure lesions [Grade 1A].*

Pelvic binder (PB) could be a “home-made” (as a bedsheet) or commercial binder (as T-POD® (Bio Cybernetics Inter-national, La Verne, CA, USA), SAM-Sling® (SAM Medical Products, Newport, OR, USA), Pelvi Binder® (Pelvic Binder Inc., Dallas, TX, USA)). Nowadays, according to ATLS guidelines PB should be used before mechanical fixation when there are signs of a pelvic ring fracture [26]. The PB right position should be around the great trochanter and the symphysis pubis to apply a pressure to reduce pelvic fracture and to adduct lower limbs in order to decrease the pelvic internal volume. Commercial pelvic binders are more effective in control pelvic bleeding than the “home-made” ones [36]. However in low resources setting or in lacking of commercial devices, “home-made” pelvic binder con be effectively and safely used.

PB is a cost-effective and a non-invasive tool that could be used by physicians and volunteers during the maneuvers aiming to stabilize a trauma patient, particularly in the immediate resuscitative period and the pre-hospital setting [1, 28, 37]. Sometimes PB can be used as bridge to definitive mechanical stabilization in those patients hemodynamically stable and mechanically unstable with no other lesions requiring treatment and with a negative CT-scan; those patients in many cases can proceed directly to definitive mechanical stabilization. Biomechanical studies on cadaver showed an effective pelvic volume reduction with an improved hemorrhage control [38–41]. These data are confirmed in vivo [42–44]. The Eastern Association for Surgery for Trauma’s pelvic trauma guidelines reporting data from the large retrospective study of Croce et al. recommended the use of PB to reduce a pelvic unstable ring [2, 42]. The use of PB alone doesn’t seem to reduce mortality [2, 42]. Authors reported a decrease in used units of blood from 17.1 to 4.9 (*p* = 0.0001) in the first 24 h, and from 18.6 to 6 after 48 h in patients treated with external fixation and PB, respectively [42]. However, comparing PB with external pelvic fixation in patients with sacroiliac fractures, Krieg et al. found a higher transfusion needs in the first 24 and 48 h in patients who underwent external fixation [43].

Some complications could occur if the binder is not removed rapidly and if it’s over-tightened: PB should not be kept for more than 24–48 h. Skin necrosis and pressure ulcerations could be increased by PB continuous application of a pressure above 9.3 kPa for more than 2–3 h [40]. As the long-term effects of pelvic binder remain unclear at present, including the potential risk of soft tissue complications from prolonged compression [45], the general recommendation is to remove pelvic binders as soon as physiologically justifiable [26], and to consider replacing binders by external pelvic fixation.

In elderly patients, even a minor trauma could cause major pelvic fractures or bleedings due to the bones fragility and the decrease in function of regulation systems as the vasospasm [46]. Lateral compression fracture pattern is more frequent, and fractures are usually not displaced. For this reason angiography seems to have more hemostatic effect than PB [44].

Even in pregnant women, the pelvis can be closed with internal rotation of the legs and PB positioning [47].

### Role of REBOA in hemodynamic unstable pelvic ring injuries

- *Resuscitative thoracotomy with aortic cross-clamping represents an acute measure of temporary bleeding control for unresponsive patients “in extremis” with exsanguinating traumatic hemorrhage. [Grade 1A]*
*- REBOA technique may provide a valid innovative alternative to aortic cross-clamping [Grade 2B].*
*- In hemodynamic unstable patients with suspected pelvic bleeding (systolic blood pressure <90 mmHg or non-responders to direct blood products transfusion), REBOA in zone III should be considered as a bridge to definitive treatment [Grade 2B].*
*- In major trauma patients with suspected pelvic trauma, arterial vascular access via femoral artery (e.g. 5Fr) introducer might be considered as the first step for eventually REBOA placement [Grade 2C].*
*- Partial-REBOA or/and intermittent-REBOA should be considered to decrease occlusion time and ischemic insult [Grade 2C].*

Resuscitative Endovascular Balloon Occlusion of the Aorta (REBOA) has emerged in recent years as alternative to emergent Resuscitative thoracotomy (RT) in hemodynamic unstable trauma patients [48–51]. The usage of REBOA and other Endo-Vascular hybrid Trauma Management (EVTM) methods is increasing worldwide in general trauma care including pelvic bleeding and now a part of the clinical praxis and guidelines in major trauma centers [6, 48–50, 52–58]. Several retrospective publications on REBOA in trauma care came lately from Japan, where REBOA has been practiced widely in the last 10–15 years but there are only few series concentrating on pelvic bleeding and REBOA [53, 57, 59, 60]. The method itself though, as a bleeding control method, has been used widely in endovascular surgery under the name Aortic Balloon Occlusion (ABO) [61–64]. REBOA is described as a “bridge to surgery” method and in pelvic bleeding as an alternative for RT with following open surgery or embolization (or both) for definitive bleeding control. REBOA can be placed in Zone I (supra-celiac or descending aorta) or Zone III (infra-renal) but preferably not in zone II (para-renal) due to risk of visceral organ ischemia. It’s been speculated that Zone III REBOA be optimal for pelvic bleeding as the ischemic insult on visceral organs is prevented and long occlusion time (4–6 h) is possible [48, 49, 52]. Trauma patients though, might have multiple injuries and unclear source of bleeding upon arrival, which makes it challenging to decide if Zone III REBOA is suitable for hemodynamic stabilization. In the majority of reported series, REBOA was placed in zone I first and then redeployed in Zone III. REBOA seems to elevate the systolic blood pressure in bleeding patients while preserving carotid and coronary flow and this data is confirmed in animal studies though there is no clear evidence of mortality benefit in the reported literature [49, 65–68]. One must consider though that the reported usage of REBOA is a mixture of different bleeding mechanism and localizations as there is not enough data of isolated pelvic bleedings reported [57, 59]. New information from the AORTA, ABOTrauma Registry and DIRECT IABO studies show preliminary beneficial results in trauma patients and some evidence that zone III REBOA as well as partial-REBOA and intermittent-REBOA might have positive effect on survival rates [54]. Zone III REBOA seems to have some benefits as time gain for surgical strategic consideration by temporary hemodynamic stabilization. It also allows time for fluid replacement as well as preparation of bleeding control procedures (surgery/angiography or hybrid procedures) [49, 52, 54, 69]. REBOA is highly dependent on a functional femoral artery access and its early establishment might be of considerable value [52, 70]. REBOA for pelvic bleeding in hemodynamic unstable patients has the advantage of being a minimal invasive procedure with less metabolic and surgical burden on the trauma patient but this is only based on expert opinion and animal experiments rather than firm data [66, 68, 71–74]. Its usage is though increasing dramatically worldwide, especially in the USA despite lack of high quality evidence and prospective trials and RCT data are needed. Two important factors to consider when using REBOA in pelvic bleeding are:

- the vascular access for REBOA, because of a functional femoral artery access must be gained first and it’s still remained to be answered who should do it and at what stage and localization should it be done. As a main rule only qualified experienced people should do this; as a counterpart however any surgeon who also does ICU or vascular should be facile at these. Lastly it must be kept in mind that having an arterial line bring some additional issues to manage: on one hand when placed it needs to be connected to ulterior lines (i.e. fluids, cable, etc.) on the other hand it also provides the most accurate blood pressure readings.

- the estimated source of bleeding is crucial for determination of REBOA zone placement. For pelvic bleeding, zone III is postulated to be preferred [48, 49, 52].

Moreover there are some major limitations to REBOA. As mentioned, REBOA is only a temporary solution and a definitive bleeding control must follow. One of the major problems of REBOA is the ischemia-reperfusion organ injury followed by multiple organ failure that might be prevented by short REBOA time, intermittent REBOA (iREBOA), Zone III REBOA and new methods as partial REBOA (pREBOA) described lately [67, 75, 76].

The insertion of REBOA is not free from risks. During maneuvers inside emergency room in a hemodynamically unstable patient, it can be time-consuming to obtain percutaneous, or US guided, or surgically exposed femoral access. Vascular injuries can be present in severe pelvic injuries or otherwise produced particularly in elderly with calcific vessels and, nowadays, most trauma surgeons reserve REBOA only in patients in extremis, with multiple sites of bleeding, as a bridge to more definitive damage control surgical techniques.

Finally, a new evolving concept is the EvndoVascular hybrid Trauma Management (EVTM) that takes into considerations early vascular arterial access, REBOA, embolization and stent-grafts for bleeding control with hybrid (Open and endovascular) procedures. This concept takes into consideration all the above in the initial treatment of trauma patients and can finally suggest to take into account the presence of a vascular surgeon in the team managing selected politraumatized patients [52, 69, 70].

### Role of Pre-peritoneal Pelvic Packing in hemodynamically unstable pelvic fractures

- *Patients with pelvic fracture-related hemodynamic instability should always be considered for pre-peritoneal pelvic packing, especially in hospitals with no angiography service [Grade 1C].*
*- Direct preperitoneal pelvic packing represents an effective surgical measure of early haemorrhage control in hypotensive patients with bleeding pelvic ring disruptions [Grade 1B].*
*- Pelvic packing should be performed in conjunction with pelvic stabilization to maximize the effectiveness of bleeding control [Grade 2A].*
*- Patients with pelvic fracture-related hemodynamic instability with persistent bleeding after angiography should always be considered for pre-peritoneal pelvic packing [Grade 2A].*
*- Pre-peritoneal pelvic packing is an effective technique in controlling hemorrhage in patients with pelvic fracture-related hemodynamic instability undergone prior anterior/C-clamp fixation [Grade 2A].*

The main source of acute retroperitoneal hemorrhage in patients with hemodynamically unstable pelvic ring disruptions is attributed to venous bleeding in 80%–90% of all cases, originating from presacral and paravesical venous plexus and from bleeding cancellous bone surfaces from sacral and iliac fractures and sacro-iliac joint disruptions [77]. Only 10%–20% of all pelvic bleeding sources are arterial [77]. Arterial bleeding may be predominant in patients with persistent hemodynamic instability after mechanical stabilization [78]. Moreover, when arterial bleeding is present, the likelihood of concomitant venous bleeding is close to 100% [46, 79]. Since venous bleeding sources are inadequately managed by angio-embolization, studies have shown that the traditional ATLS-guided management of hemodynamically unstable pelvic ring injuries with angio-embolization results in poor patient outcomes with high post-injury mortality rates greater than 40% [80, 81]. The notion of a mainly venous retroperitoneal bleeding source in pelvic fractures provides the main rationale for pelvic packing for acute surgical hemorrhage control [4, 82].

Pre-peritoneal pelvic packing (PPP) has become a commonly used technique to control bleeding in hemodinamically unstable pelvic fractures in recent years. PPP has been reported to be a quick and easy-to-perform technique [4, 79] and it could be accomplished both in the emergency department (ED) and the operating room [4]. In experienced hands it can be completed with a minimal operative blood loss in less than 20 min [79, 83]. Since its first description by Hannover and Zurich groups in patients with pelvic ring injuries, outcomes have been improved by early surgical “damage control” intervention, including temporary external stabilization of unstable pelvic fractures, transabdominal pelvic packing, and surgical bleeding control [84–86].

More recently, the concept of “direct” preperitoneal pelvic packing (PPP) was described in Denver using a distinct surgical technique by a separate suprapubic midline incision that allows a direct retroperitoneal approach to the space of Retzius [83]. The modified PPP technique allows for more effective packing within the concealed preperitoneal space with three laparotomy pads for each side of the bladder in the retroperitoneal space packed below the pelvic brim towards the iliac vessels [79, 83, 87], without the necessity of opening the retroperitoneal space [82, 83]. With this technique, a midline laparotomy can be performed through a separate incision proximal to the suprapubic approach, if indicated for associated intra-abdominal injuries [88]. The separate incision technique has been shown to be safe with regard to preventing cross-contamination from intra-abdominal injuries to the retroperitoneal space and thereby decreasing the risk of postoperative infections after pelvic packing and subsequent pelvic fracture fixation [88]. PPP revision should be done within 48–72 h.

Retrospective observational studies revealed that the implementation of standardized multidisciplinary clinical guidelines that include early surgical management with pelvic external fixation and direct PPP for hypotensive patients with hemodynamical and mechanical unstable pelvic ring injuries led to a significant decrease of transfused blood products and to a significantly decreased post-injury mortality [5, 6, 87]. More recent observational studies confirmed the notion that extraperitoneal pelvic packing is a safe and fast procedure associated with a significantly reduced mortality in hemodynamically unstable patients with pelvic fractures, compared to patients managed by conventional measures without pelvic packing [89–91].

In hemodynamically and mechanically unstable pelvic fractures, PPP should be performed along with external fixation [46, 56, 79]. Cothren et al. showed that external fixation and PPP could be sufficient to control bleeding in severely injured patients with pelvic fractures, reporting that only 13% of patients required a subsequent angioembolization for an arterial blush [82]. In very sick patients, pelvic ring stabilization can be rapidly obtained by pelvic binder, with posterior compression using rolled surgical towels under the binder in sacro-iliac disruption [92].

Subsequent (secondary) angioembolization is recommended in the selected cohort of patients with ongoing hemorrhage and/or transfusion requirements after the pelvic packing procedure [4, 29, 56, 79, 87, 93]. The need for angioembolization following PPP has been reported to be between 13 and 20% [56, 87, 91]. However, Totterman et al. reported that 80% of patients who underwent PPP had positive findings for arterial injury at angiography [94].

PPP has been proposed as an alternative to angiography [79, 87, 91, 93]. Some papers [87, 91, 93] compared the use of PPP vs. Angioembolization. In a recent a prospective quasi-randomized trial Li et al. [91] showed that time-to-procedure and procedure time were significantly shorter in the PACK group than in the ANGIO one. The need for packed red cells in the first 24 h after procedure, the need for complementary procedures (angiography or PPP), mortality rates did not differ between the two groups [91]. Present guidelines recommend considering angiography and PPP as complementary procedures.

### Role of external pelvic fixation in hemodynamic unstable pelvic ring injuries

*- External pelvic fixation provides rigid temporary pelvic ring stability and serves as an adjunct to early haemorrhage control in hemodynamically unstable pelvic ring disruptions [Grade 1A].*
*- External pelvic fixation is a required adjunct to preperitoneal pelvic packing to provide a stable counterpressure for effective packing [Grade 2A].*
*- Anterior “resuscitation frames” through iliac crest or supra-acetabular route provide adequate temporary pelvic stability in APC-II/-III and LC-II/-III injury patterns. A posterior pelvic C-clamp can be indicated for hemorrhage control in “vertical shear” injuries with sacroiliac joint disruptions [Grade 2A].*
*- Pelvic C-clamp application is contraindicated in comminuted and transforaminal sacral fractures, iliac wing fractures, and LC-type pelvic ring disruptions [Grade 2B].*

The biomechanics of pelvic ring injuries and the underlying trauma mechanism dictate the need for external fixation [58, 95]. Pelvic ring disruptions in hemodynamically unstable patients should be temporarily stabilized to prevent further hemorrhage and to support measures of hemorrhage control, including angiography and pelvic packing [28, 46, 58, 96, 97]. The rationale for acute external pelvic fixation consists of (1) reducing the intrapelvic volume in “open book” equivalent injuries to decrease the retroperitoneal bleeding space, and (2) to provide a stable counter-pressure to the “packed” lap sponges for effective pelvic packing. For example, pelvic packing is not effective in absence of adequate counterpressure by posterior pelvic elements, which requires external fixation for unstable pelvic ring disruptions [56, 87, 98]. The technical aspects of decision-making for the modality of “damage control” external fixation for unstable pelvic ring injuries have been described elsewhere [58]. In essence, the indication and technique of pelvic external fixation can be guided by the Young & Burgess fracture classification [58, 99]. Unstable antero-posterior compression (APC-II/APC-III) and lateral compression injuries (LC-II/LC-III) injuries are ideally managed by anterior resuscitation frames, using iliac crest or supra-acetabular Schanz pin application. While the iliac crest route is technically less demanding and allows a faster “damage control” application, the pull-out resistance of Schanz pins in the iliac crest is very low and therefore associated with a higher risk of failure of reduction and fixation. In contrast, supra-acetabular frames require diligent pin placement under radiographic control using a C-arm, however, these frames have a very high pull-out resistance due to the solid supra-acetabular surgical corridor [58]. In contrast to rotationally unstable APC and LC-type injuries, vertically unstable pelvic ring disruptions, such as “vertical shear” (VS) injuries, are best stabilized by a posterior C-clamp [84, 86, 100–103]. Of note, the trauma surgeon must be aware of inherent risks and potential technical complications using the C-clamp due to the learning curve and required experience for safe application [104, 105]. Contraindications for the application of a pelvic C-clamp include comminuted and transforaminal sacral fractures, fractures of the iliac wing, and lateral compression-type injuries [58]. For these reasons, C-clamp is not used in many trauma centers.

### Role of Angioembolization in hemodynamic unstable pelvic fractures

- *Angioembolization is an effective measure of haemorrhage control in patients with arterial sources of retroperitoneal pelvic bleeding [Grade 1A].*
*- CT-scan demonstrating arterial contrast extravasation in the pelvis and the presence of pelvic hematoma are the most important signs predictive of the need for angioembolization [Grade 1C].*
*- After pelvic stabilization, initiation of aggressive hemostatic resuscitation and exclusion of extra-pelvic sources of blood loss, patients with pelvic fractures and hemodynamic instability or evidence of ongoing bleeding should be considered for pelvic angiography/angioembolization [Grade 2A].*
*- Patients with CT-scan demonstrating arterial contrast extravasation in the pelvis may benefit from pelvic angiography/angioembolization regardless of hemodynamic status [Grade 2A].*
*- After extra-pelvic sources of blood loss have been ruled out, patients with pelvic fractures who have undergone pelvic angiography with or without angioembolization, with persisting signs of ongoing bleeding, should be considered for repeat pelvic angiography/angioembolization [Grade 2B].*
*- Elderly patients with pelvic fractures should be considered for pelvic angiography/angioembolization regardless of hemodynamic status [Grade 2C].*

Since the 1980s, percutaneous trans-catheter angioembolization has been shown to represent an effective non-surgical measure of acute bleeding control in hemodynamically unstable pelvic fractures [106–109]. Most published clinical guidelines recommend the use of early angioembolization, in conjunction with external pelvic fixation if indicated, as the main measure of acute bleeding control [10, 46, 93, 110–117]. As a counterpart it is important to consider a number of factors that are critical to decision-making. The exclusive use of angioembolization has been associated with a high mortality in patients with bleeding pelvic fractures [118], which was significantly reduced by application of a combined protocol with initial preperitoneal pelvic packing and subsequent (secondary) angioembolization, if indicated [28, 56, 79, 86, 89]. It has been estimated that 85% of pelvic bleeding originates from bone, soft tissues, or major venous structures [2]. In addition, as many as 90% of patients with unstable pelvic fractures will have significant associated injuries. Bleeding in the abdomen, chest, or extremities will contribute to shock and may require more urgent control than the pelvic bleeding. Thus, the fundamental management principles include aggressive hemostatic resuscitation, bony stabilization of the pelvis, and identification and management of extrapelvic bleeding. Management guidelines that emphasize these principles demonstrate improved outcomes [6, 16, 46, 116]. Pelvic Angiography/Angioembolization (AG/AE) is expected to benefit only a small minority of patients, and therefore should be employed once extrapelvic and non-arterial sources of bleeding are controlled [2]. Arterial contrast extravasation seen on CT scan is a good indicator of the need for pelvic AG/AE [114]. In contrast, fracture pattern alone has not been predictive of who will require angiography [119]. Pelvic AG/AE is very effective in controlling hemorrhage. However, some patients will continue to bleed and repeat AG/AE has been found to be an effective strategy [115]. Elderly patients have been found to require AG/AE more frequently than younger adults, regardless of apparently normal hemodynamics at presentation, even in mechanical stable-low risk fractures. Therefore, AG/AE should be considered in these patients even when there is low suspicion of pelvic bleeding [120].

### Indications for definitive surgical fixation of pelvic ring injuries

- *Posterior pelvic ring instability represents a surgical indication for anatomic fracture reduction and stable internal fixation. Typical injury patterns requiring surgical fixation include rotationally unstable (APC-II, LC-II) and/or vertically unstable pelvic ring disruptions (APC-III, LC-III, VS, CM) [Grade 2A].*
*- Selected lateral compression patterns with rotational instability (LC-II, L-III) benefit from adjunctive, temporary external fixation, in conjunction to posterior pelvic ring fixation [Grade 2A].*
*- Pubic symphysis plating represents the modality of choice for anterior fixation of “open book” injuries with a pubic symphysis diastasis > 2.5 cm (APC-II, APC-III) [Grade 1A].*
*- The technical modality of posterior pelvic ring fixation remains a topic of debate, and individual decision-making is largely guided by surgeons’ preference. Spinopelvic fixation has the benefit of immediate weight bearing in patients with vertically unstable sacral fractures [Grade 2C].*
*- Patients hemodynamically stable and mechanically unstable with no other lesions requiring treatment and with a negative CT-scan can proceed directly to definitive mechanical stabilization [Grade 2B].*

Pelvic ring injuries with rotational or vertical instability require surgical fixation with the goal of achieving anatomic reduction and stable fixation as a prerequisite for early functional rehabilitation. There is general consensus that pelvic ring disruptions with instability of posterior elements require internal fixation [95, 121]. Trauma mechanism-guided fracture classifications, including the widely used Young & Burgess system, provide guidance for surgical indications for pelvic fracture fixation [58, 122]. For example, stable fracture patterns, such as antero-posterior compression type 1 (APC-I) and lateral compression type 1 (LC-I) injuries are managed non-operatively, allowing functional rehabilitation and early weight bearing [123, 124]. In contrast, rotationally unstable APC-II/APC-III (“open book”) injuries and LC-II fracture patterns (“crescent fracture”), as well as rotationally and vertically unstable LC-III (“windswept pelvis”), “vertical shear” (VS), and “combined mechanism” (CM) fracture patterns require definitive internal fixation [123, 124]. Multiple technical modalities of surgical fixation have been described, including open reduction and anterior plating of pubic symphysis disruptions, minimal-invasive percutaneous iliosacral screw fixation for unstable sacral fractures and iliosacral joint disruptions, plating of iliac wing fractures, and spino-pelvic fixation (named “triangular osteosynthesis” in conjunction with iliosacral screw fixation) or tension band plating for posterior pelvic ring injuries, including vertically unstable sacral fractures [125–133]. In addition, selected lateral compression (LC) type injuries are occasionally managed with temporary adjunctive external fixators for 6 weeks post injury, to protect from rotational instability of the anterior pelvic ring [58, 134]. Minimal invasive anterior “internal fixators” have been recently described as an alternative technical option [135]. The ultimate goal of internal fixation of unstable pelvic ring injuries is to allow early functional rehabilitation and to decrease long-term morbidity, chronic pain and complications that have been historically associated with prolonged immobilization [136, 137].

### Ideal time-window to proceed with definitive internal pelvic fixation

*- Hemodynamically unstable patients and coagulopathic patients “in extremis” should be successfully resuscitated prior to proceeding with definitive pelvic fracture fixation [Grade 1B].*
*- Hemodynamically stable patients and “borderline” patients can be safely managed by early definitive pelvic fracture fixation within 24 h post injury [Grade 2A].*
*- Definitive pelvic fracture fixation should be postponed until after day 4 post injury in physiologically deranged politrauma patients [Grade 2A].*

The timing of definitive internal fixation of unstable pelvic ring injuries remains a topic of debate [138–145]. Most authors agree that patients in severe traumatic-hemorrhagic shock from bleeding pelvic ring disruptions are unlikely candidates for early definitive pelvic fracture fixation, due to the inherent risk of increased mortality from exsanguinating hemorrhage and the “lethal triad” of coagulopathy, acidosis and hypothermia [22, 146]. A prospective multicenter cohort study revealed a significantly increased extent of blood loss and increased interleukin (IL-6 and IL-8) serum levels, reflective of an exacerbated systemic inflammatory response, in politrauma patients who underwent early pelvic fracture fixation on the first or second day post injury [147]. The early timing and short duration of initial pelvic stabilization revealed to have a positive impact on decreasing the incidence of multiple organ failure (MOF) and mortality [148]. Furthermore, post-injury complication rates were shown to be significantly increased when definitive pelvic ring fixation was performed between days 2 and 4, and decreased when surgery was delayed to days 6 to 8 post injury [149]. Many authors concur with the traditional concept of initial “damage control” external fixation of hemodynamically unstable pelvic ring injuries, and delayed definitive internal fixation after day 4, subsequent to successful resuscitative measures [28, 41, 58, 95, 118, 150–152]. The use of such definitions and classification systems can provide guidance for future stratification of unstable politrauma patients with pelvic ring injuries requiring “damage control” resuscitative measures compared to stable or “borderline” patients who may be safely amenable to early total care by definitive pelvic fracture fixation [141, 146]. In this regard, multiple observational cohort studies from the orthopedic trauma group at MetroHealth in Cleveland have shown that early pelvic fracture fixation in stable or borderline resuscitated patients within 24 h of admission reduces the risk of complications and improves outcomes [139, 141, 144, 145]. Recently, a new definition of politrauma has been proposed by an international consensus group, which is based on injury severity and derangement of physiological parameters [153]. This new politrauma definition in conjunction with recently established grading systems [141] may provide further guidance towards the “ideal” timing of definitive pelvic fracture fixation, pending future validation studies.

### Damage Control Orthopedics in Severe Head Injuries

Severe head injuries are common in politrauma patients with concomitant pelvic injuries. No definitive guidelines exist regarding severe head injuries and pelvic fixation. One of the main issues is that pelvic fracture associated bleeding and consequent coagulopathy leads to a deterioration of the head injury through secondary bleeding and subsequent progression of hemorrhagic contusions in a risky vicious circle. For these reasons the acute definitive hemorrhage control and prevention and prompt reversal of coagulopathy is essential. Careful monitoring of brain injuries, potential early re-scanning with perfusion CT-scan is helpful. In the major part of the trauma centers patients are treated according to the indications of the neurosurgery team [150]. On one hand several articles suggested that early fracture fixation might be deleterious in patients with brain injury especially if old-aged, on the other hand however some trials didn’t confirm these concerns suggesting that outcomes are worse in patients who do not have early skeletal stabilization [44, 154–156]. Usually neurosurgeons are very concerned for the possible additional brain injury deriving from blood pressure fluctuations during orthopedic fixative surgery [150]. This in general leads to several doubts and additional delay to let the patients being considered suitable for operating room [150]. The potential benefit of damage control orthopedics interventions and the minimal physiologic insult of placing an external fixator allows for almost all patients with closed head injuries to be appropriate for at least external fixation [150]. However no definitive indications can be obtained from the literature.

### Morbidity, mortality and outcomes

Complications with important functional limitations are present especially in patients with open PT who may have chronic sequelae as fecal and urinary incontinence, impotence, dyspareunia, residual disability in physical functions, perineal and pelvic abscess, chronic pain and vascular complications as embolism or thrombosis [1, 3].

The majority of deaths (44.7%) occurred on the day of trauma and the main factors that correlate with mortality are increasing age, ISS, pelvic ring instability, size and contamination of the open wound, rectal injury, fecal diversion, numbers of blood units transfused, head Abbreviated Injury Scale (AIS), admission base deficit [3, 5].

Lastly, a recent study reported the impact given by the multidisciplinary approach resulting in an improvement in performance and in patient outcomes [5]. At first a defined decision making algorithm reduce significantly (*p* = 0.005) the time from hospital arrival and bleeding control in the theatre with PPP [5]. Furthermore the definition of a massive hemorrhage protocol reduced significantly the use of liquids administered prior blood transfusions and rationalized the use of packed red cells and fresh frozen plasma (ratio 2:1) starting within the first hours following injury [5]. Moreover a dedicated pelvic orthopedic surgeons can improve (*p* = 0.004) the number of patients that undergoing definitive unstable pelvic fractures repair with a consequently improvement in outcome [5]. Similar data about the importance of the adherence to defined guidelines have been reported by Balogh et al. [16] and recently confirmed by the multi-institutional trial by Costantini et al. [10].

## Conclusions

the management of pelvic trauma must keep into consideration the physiological and mechanical derangement. Critical and operative decisions can be taken more effectively if both anatomy of injury and its physiological and mechanical effects are considered.

## Abbreviations

ABO: Aortic Balloon Occlusion
AE: Angioembolization
AG: Angiography
AIS: Abbreviated Injury Score
APC: Antero Posterior Compression
ATLS: Advanced Trauma Life Support
BD: Base Deficit
BPM: Beat Per Minute
CM: Combined Mechanism
CT: Computed Tomography
DSA: Digital Subtraction Angiography
ED: Emergency Department
E-FAST: Extended-Focused Assessment with Sonography for Trauma
EVTM: Endovascular Trauma Management
ICU: Intensive Care Unit
IREBOA: Intermittent Resuscitative Endo Vascular Balloon Occlusion
ISS: Injury Severity Score
LC: Lateral Compression
LE: Level of Evidence
MOF: Multi-Organ Failure
NOM: Non-Operative Management
OM: Operative Management
PB: Pelvic Binder
PPP: Pre-peritoneal Pelvic Packing
PREBOA: Partial Resuscitative Endo Vascular Balloon Occlusion
PT: Pelvic Trauma
PXR: Pelvic X-ray
RCT: Randomized Controlled Tria
REBOA: Resuscitative Endo Vascular Balloon Occlusion
ROTEM: Rotational Thromboelastometry
RUG: Retrograde Urethrogram
TEG: Thromboelastography
VS: Vertical Shear
WSES: World Society of Emergency Surgery
